# Dysplasie fibreuse ethmoïdo-frontale: à propos d’un cas

**DOI:** 10.11604/pamj.2021.38.385.27561

**Published:** 2021-04-20

**Authors:** Zeineb Ayadhi, Aymen Ben Youssef, Nesrine Hamroun

**Affiliations:** 1 Service d’Otorhinolaryngologie et Chirurgie Cervico-Faciale, Hôpital Régional de Menzel Bourguiba, Tataouine, Tunisie

**Keywords:** Dysplasie fibreuse, tomodensitométrie, chirurgie, à propos d’un cas, Fibrous dysplasia, computed tomography, surgery, case report

## Abstract

La dysplasie fibreuse est une maladie osseuse génétique et rare du sujet jeune. Sa physiopathologie implique des mutations génétiques à l´origine d´un défaut de maturation osseuse avec prolifération fibreuse médullaire. L´extrémité céphalique est atteinte dans un tiers des cas. Nous en présentons un cas de localisation ethmoïdo-frontale traité chirurgicalement. Il s´agissait d´un homme âgé de 31ans qui a consulté pour des céphalées holocrâniennes chroniques. L´imagerie par tomodensitométrie a objectivé un volumineux processus expansif ethmoïdo-frontal bilatéral hyperdense. L´indication chirurgicale a été retenue devant les signes fonctionnels et les données de l´imagerie. L´examen anatomopathologique des fragments osseux a confirmé le diagnostic de dysplasie fibreuse. La dysplasie fibreuse est une maladie rare avec une évolution lente. En absence de consensus thérapeutique, la chirurgie reste le traitement de référence pour les formes unifocales.

## Abstract (en)

Fibrous dysplasia is a genetic and rare bone disorder affecting the young subject. Its pathophysiology involves genetic mutations leading to defective skeletal development with fibrous and medullary proliferations. Cephalic extremity is involved in one third of cases. We here report a case of surgically treated fibrous dysplasia of the frontal and ethmoid sinuses. The study involved a 31-year-old man presenting with chronic holocranial headaches. Computed tomography (CT) scan showed voluminous bilateral hyperdense expansive process in the frontal and ethmoid sinuses. Surgery was performed on the basis of functional signs and imaging data. Anatomopathological examination of bone fragments confirmed the diagnosis of fibrous dysplasia. Fibrous dysplasia is rare and characterized by slow disease progression. In the absence of a consensus on therapy, surgery remains the treatment of choice for unifocal forms.

## Introduction

La dysplasie fibreuse (DF) est une affection osseuse sporadique et rare du sujet jeune, caractérisée par un défaut de maturation osseuse localisé à un ou plusieurs sites osseux [1]. Sa physiopathologie implique un ensemble de mutations génétiques qui génèrent une hyperactivité ostéoclastique avec prolifération fibreuse médullaire [2]. Ses localisations crâniofaciales se manifestent le plus souvent par des tuméfactions osseuses indolores et asymétriques ainsi que par des troubles fonctionnels ophtalmologiques, otologiques et/ou neurologiques. Nous rapportons le cas d´une dysplasie fibreuse ethmoïdo-frontale chez un patient de 31 ans traité chirurgicalement.

## Patient et observation

Un homme de 31 ans sans antécédents pathologiques, a consulté pour des céphalées holocrâniennes évoluant depuis un an, à maximum frontal, paroxystiques, non améliorées par les antalgiques usuels, sans autres signes rhinologiques ni otologiques associés. Le patient présentait un élargissement de la pyramide nasale. Le reste de l´examen oto-rhino-laryngologique et somatique était sans particularités, notamment l´endoscopie nasale.

Une tomodensitométrie cérébrale et du massif facial ont objectivé un volumineux processus expansif ethmoido-frontal bilatéral hyperdense, hétérogène et bien limité avec épaississement de la paroi postérieure du sinus frontal. Devant les signes fonctionnels, l´atteinte de la table interne du sinus frontal et l´extension au toit de l´orbite et à la lame papyracée nous avons retenu l´indication chirurgicale.

**Figure 1:**
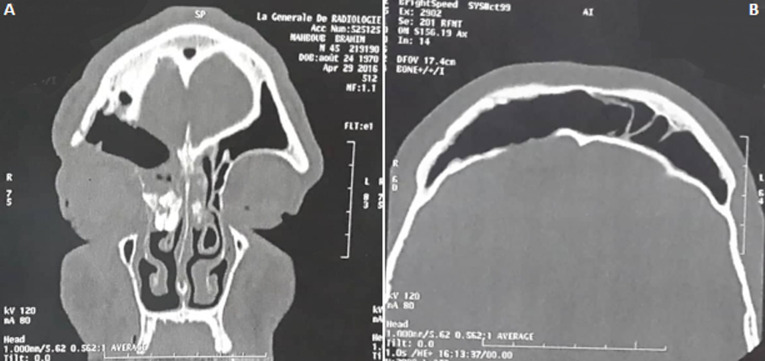
A) scanner du massif facial en coupe sagittale montrant la cranialisation du sinus frontal; B) scanner du massif facial en coupe coronale montrant la cranialisation du sinus frontal

**Figure 2:**
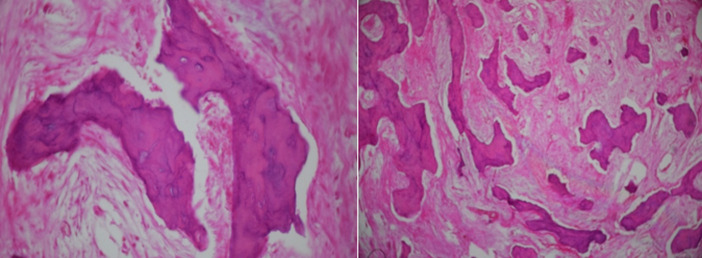
aspect histologique des fragments osseux étudiés avec lamelles osseuses non bordées de couronne ostéoblastique reposant sur un fond fibreux

A travers une incision paralatéronasale étendue en voie de Jacques nous avons effectué un évidement ethmoïdal droit et frontal bilatéral à l´aide d´un ostéotome, avec résection de la paroi supérieure de l´orbite et une crânialisation du sinus frontal (Figure 1A, Figure 1B). La tumeur était hétérogène, solide et légèrement calcifiée. Les suites opératoires ont été marquées par une déglobulisation de 4g/dl, ayant nécessité une transfusion sanguine. L´étude anatomopathologique des fragments osseux, après décalcification et coloration à l´hématoxyline - éosine, a mis en évidence une prolifération tumorale faite de lamelles osseuses sur un fond fibreux par endroits riche en collagène. Les lamelles osseuses n´étaient pas bordées en périphérie d´une couronne ostéoblastique (Figure 2). Cette prolifération tumorale arrivait par places sous une muqueuse respiratoire régulière.

**Figure 3:**
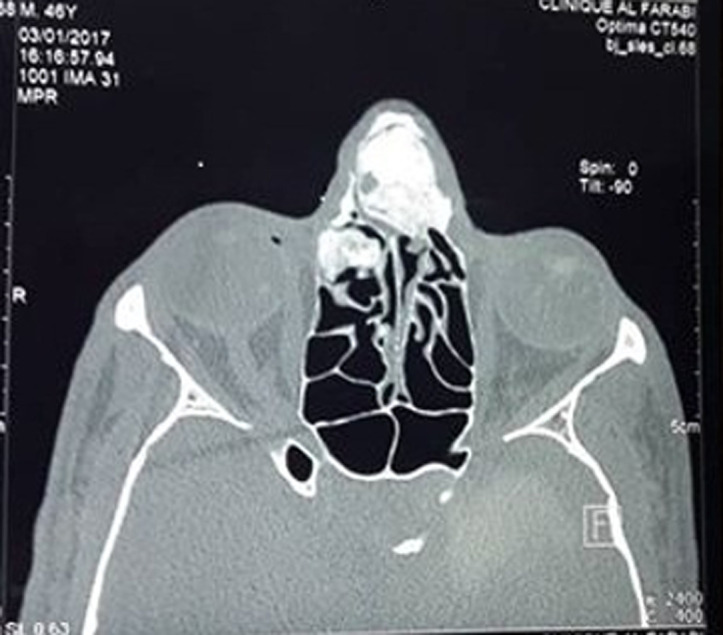
coupe transversale passant par la pyramide nasale montrant le reliquat tumoral en place

Une tomodensitométrie de contrôle, faite huit mois après la chirurgie, a objectivé un reliquat tumoral ethmoïdal droit lysant la lame papyracée droite, que nous avons réopéré par la même voie d´abord à minima. La localisation tumorale au niveau des os propres du nez et la branche montante du maxillaire a été respectée devant l´absence de retentissement fonctionnel et social pour le patient et le risque esthétique majeur qu´aurait entrainé son exérèse (Figure 3). L´évolution était favorable avec un recul d´un an.

## Discussion

La DF est une lésion bénigne sporadique congénitale qui représente 1% de toutes des tumeurs osseuses et 7% des tumeurs osseuses bénignes [3]. Rapportée pour la première fois par Von Recklinghausen en 1891 chez des patients porteurs de neurofibromatose, elle a été présentée en détails par McCune and Brunch en 1937 et dénommée par Lichtenstein en 1938 [4]. Sa prévalence, inférieure à 1/2000 est probablement sous-estimée du fait des formes asymptomatiques. La DF touche les deux sexes avec une prédominance féminine (sex ratio 3/1) et l´âge au diagnostic est le plus souvent compris entre 5 et 30 ans avec une moyenne de 28,6 ans [5].

Due essentiellement à un défaut de maturation des ostéoblastes, la DF résulte d´une mutation somatique hétérozygote du gène GNAS survenant à un stade précoce du développement embryonnaire. Ce gène est situé sur le locus 20q13 code pour la sous-unité alpha de la protéine G stimulatrice. Cette mutation post-zygotique précoce génère une mosaïque somatique dans laquelle les cellules mutées et les cellules normales coexistent dans les segments osseux affectés [6]. Cette mutation conduit à une augmentation de l´activité de l´adénylcyclase avec synthèse accrue de l´AMPc intracellulaire, activateur enzymatique. Dans l´os, cette hyperproduction se traduit par l´inhibition de la différenciation et de la prolifération des cellules stromales à l´origine des différents phénotypes cellulaires (ostéoblastes, adipocytes et cellules hématopoïétiques) par modification de leur cytosquelette.

L´os normal est ainsi remplacé par une matrice structurellement et fonctionnellement anormale avec fibrose extensive et perte locale de l´hématopoïèse. D´autres types cellulaires sont également affectées par cette mutation : les mélanocytes et les cellules endocrines (gonades, thyroïde, hypophyse, surrénales). De plus, une augmentation de l´expression de l´interleukine 6 qui inhibe la formation osseuse et stimule la formation et l´activation des ostéoclastes a été décrite dans la DF, entrainant une résorption osseuse accrue touchant l´ethmoïde (72%), le sphénoïde (43%), l´os frontal (33%), le maxillaire (24%), et plus rarement les os temporal pariétal et occipital. Selon le siège on peut observer plusieurs complications: compression des structures vasculo-nerveuses de la base du crane à l´origine d´une baisse de l´acuité visuelle, d´une paralysie nerveuse et céphalées tenaces [7].

A la tomodensitométrie, l´atteinte osseuse peut être lytique (20 à 30% des cas), condensante (20 à 30%), ou mixte (40 à 50%), associant des zones ossifiées et des zones kystiques. Bien que très évocateurs, ces aspects TDM peuvent prêter à confusion avec: le fibrome ossifiant dont les lésions siègent volontiers au niveau de l´os maxillaire, l´ostéome qui touche volontiers l´homme entre 40 et 50 ans, avec des lésions hétérogènes au scanner, l´ostéoblastome, tumeur de l´homme jeune et qui donne des déformations osseuses douloureuses très évocatrices; le méningiome en plaque avec épaississement et une calcification des méninges au contact d´un foyer osseux dense et spiculé, ou la maladie de Paget avec un aspect floconneux de la voute crânienne [8].

Les lésions de DF ont le plus souvent une croissance lente et indolente. La transformation maligne est décrite dans 0,4 à 4% des cas. Par ordre de fréquence on observe les ostéosarcomes, les fibrosarcomes et les chondrosarcomes. Les facteurs prédictifs de transformation ne sont pas connus [9]. Le traitement de référence des DF crâniofaciales est chirurgical et avec un taux de récidive postopératoire estimé à 25%. L´indication chirurgicale doit être retenue en cas de douleurs rebelles aux traitements médicaux, de préjudice esthétique, ou signes compressifs menaçants. Dans les formes quiescentes, la surveillance et le traitement médical sont à privilégier [10].

En cas de douleurs osseuses résistantes aux antalgiques et aux anti-inflammatoires ou de lésions osseuses à risque de fracture sans indication chirurgicale, des médicaments antirésorptifs comme le pamidronate (bisphosphonate de seconde génération et inhibiteur de l´activité ostéoclastique) peuvent être proposés à visée antalgique [11]. Le pamidronate est la molécule de choix en première intention à raison de 180 mg dose totale tous les 6 mois, répartie sur 2 ou 3 jours pour un adulte et 1 mg/kg/jour pendant 3 jours tous les 6 mois pour un enfant. Un supplément en calcium (1 g/jour) et vitamine D3 (800 UI/jour) en association aux bisphosphonates est recommandé car ces patients présentent fréquemment une carence en vitamine D qu´il faudra rechercher. Quelques effets indésirables ont été décrits : des douleurs osseuses prédominant sur les sites de fibrodysplasie, une fièvre modérée (maximum 38,5) ou une hypocalcémie (minimum 2,05 mmol) [12]. L´abstention thérapeutique est de mise en l´absence de retentissement esthétique et fonctionnel.

## Conclusion

La dysplasie fibreuse est une dystrophie osseuse génétiquement déterminée, non héréditaire, rare avec une évolution lente. Le diagnostic est facile et repose sur l´imagerie. Cependant, pas de consensus concernant le traitement. La surveillance des patients doit être prolongée vu le risque de dégénérescence sarcomateuse des lésions. Les progrès sont attendus de la thérapie génique afin d´éviter une chirurgie parfois mutilante.
